# Digital health technologies and artificial intelligence for shared decision making in acute pain management: recent advances and perspectives

**DOI:** 10.1016/j.bjao.2026.100865

**Published:** 2026-09-30

**Authors:** Dalia M. Aljohani, Shouq S. Alghamdi, Patrice Forget

**Affiliations:** 1 Department of Anesthesia Technology, Prince Sultan Military College of Health Sciences, Dhahran, Saudi Arabia; 2 Academic Primary Care, Institute of Applied Health Sciences, University of Aberdeen, Aberdeen, UK; 3 Pain and Opioids After Surgery European Society of Anaesthesiology and Intensive Care Research Group, Brussels, Belgium; 4 Epidemiology Group, Institute of Applied Health Sciences, School of Medicine, Medical Sciences and Nutrition, University of Aberdeen, Foresterhill, Aberdeen, UK; 5 Department of Anaesthesia, NHS Grampian, Aberdeen, UK

**Keywords:** acute pain, artificial intelligence, digital health technologies, pain management, shared decision making

## Abstract

Acute pain management is shifting away from traditional paternalistic models towards more collaborative, patient-centred approaches that emphasise shared decision making and the use of digital health technologies. Here, we review recent advances in digital health technologies and artificial intelligence (AI) that support shared decision making, including interactive decision aids, video narratives, and AI-assisted tools. These innovations have the potential to optimise analgesia, reduce the risks associated with opioids and opioid-related harms, and enhance patient autonomy across surgical, emergency, and primary care settings.

Although emerging evidence shows promise in improving prescribing efficiency and increasing patient satisfaction, there are still significant barriers to implementation. These include resource limitations, challenges in selecting appropriate tools, and the necessity for robust validation of AI systems. Herein, we critically evaluate the current evidence, discuss practical strategies for implementation, and provide recommendations for integrating these technologies into routine clinical practice.

Pain is recognised as one of the major global health burdens.^1^ Anyone, regardless of age, race, or other patient characteristics, can experience it. It is one of the most common reasons for seeking healthcare and remains a significant challenge in surgical settings. Despite advancements in pain management and analgesic strategies, at least one in three adults experience moderate-to-severe pain on the first day after discharge, with this rate increasing to 58% in the weeks that follow the procedure.^2^

Acute pain is defined as ‘the normal, predicted physiological response to an adverse chemical, thermal, or mechanical stimulus… associated with surgery, trauma, and acute illness’.^3^ In primary care, the most common instances of acute pain are related to musculoskeletal issues,^4^^,^^5^ and in secondary care, it is a primary reason for visits to the emergency department.^6^ Pain management, in particular postoperative pain, is receiving increasing attention.^7^^,^^8^

Traditionally, acute pain management has followed paternalistic models, which are clinician-led approaches that can overlook patients’ values, preferences, risk tolerance, and previous experiences with analgesics. These factors significantly influence treatment adherence, satisfaction, and outcomes.^9^^,^^10^ Patient-centred care (PCC), which integrates clinical expertise with patient values and preferences, represents a fundamental shift from this model. Shared decision making (SDM),^11^ where patients are positioned as active partners in treatment decisions, has emerged as a promising framework for delivering care.^12^^,^^13^ Although PCC and SDM are increasingly recognised in principle, their implementation in clinical practice remains limited.^14^^,^^15^

In acute pain settings, SDM has attracted particular interest, especially where there is a need to balance effective analgesia with concerns about opioid use, individualised care, patient autonomy, and the need for opioid stewardship.^9^ The implementation of PCC principles, including SDM, has been linked to higher patient satisfaction, better treatment adherence, and reduced healthcare utilisation across various clinical settings, although much of the recent literature focuses specifically on opioid prescribing decisions.^16^

At the same time, digital health technologies and artificial intelligence (AI) offer new opportunities to support SDM through interactive decision aids, predictive analytics, and personalised pain assessment tools.^17^^,^^18^ The convergence of SDM and digitalisation is particularly relevant in the context of the opioid crisis, primarily in North America, where balancing effective analgesia with opioid stewardship has become a critical clinical and public health priority.^19^

However, integrating these concepts into routine clinical practice raises important questions about effectiveness, safety, equity, and feasibility of implementation. Herein, we provide an evidence-based perspective on recent advances in digital health and AI for SDM in acute pain management. We review the current evidence, discuss the advantages and limitations of various approaches, and offer practical recommendations for implementation (Table 1).^20^, ^21^, ^22^, ^23^, ^24^, ^25^, ^26^, ^27^, ^28^, ^29^, ^30^, ^31^, ^32^, ^33^, ^34^, ^35^

**Table 1:** Key studies on digital health technologies and artificial intelligence for SDM in acute pain management. 3D, three-dimensional; AI, artificial intelligence; AI-PCA, artificial intelligence–enhanced patient-controlled analgesia; AQI, Analgesia Quality Index; IQR, interquartile range; ML, machine learning; NRS, numerical rating scale; SDM, shared decision making.

Author, yr	Study design	Population	Tool type	Intervention/SDM approach	Key outcomes	Limitations
**SDM tools in acute pain management**						
Manoharan and colleagues,^20^ 2023	Mixed-methods: qualitative (interviews) and quantitative (electronic health records)	Patients with complex pain histories, psychological distress, and preoperative opioid use, undergoing surgery (*n*=26)	Multidisciplinary SDM service	Multidisciplinary transitional perioperative pain service incorporating SDM, pain specialists, and psychiatric support	Reduced opioid use, improved post-surgical recovery, and high patient satisfaction with SDM approach	Small sample size; single centre; recall bias (interviews conducted 6 months to 3 yr after treatment)
Engel-Rebitzer and colleagues,^21^ 2023	Secondary analysis of an RCT	Adults aged 18–70 yr presenting to the emergency department with musculoskeletal back pain or renal colic (*n*=433)	Video narrative (culturally concordant)	Video narratives about opioids featuring storytellers matched by gender, race, or family addiction history	Watching videos with demographically concordant storytellers improved recall of pain management information the day after discharge (86% *vs* 73%; *P*=0.02). Individuals who viewed a video about a family history of addiction reported being more involved in choosing their course of treatment than those who did not watch (7.6 *vs* 6.8 on a 10-point scale; *P*=0.04).	Single centre; heterogeneous pain conditions; self-reported data subject to recall bias; limited diversity of study participants
Dolan and colleagues,^22^ 2022	Qualitative study nested within an RCT	Emergency department patients with musculoskeletal or renal colic pain, diverse in age, gender, race, and opioid risk levels (*n*=36 interviews, single centre)	Probabilistic risk tool+video narrative	Probabilistic opioid risk tool and narrative-enhanced probabilistic risk tool (short video stories)	Both tools prompted meaningful patient reflections on treatment choices; success was influenced by self-identity, previous experiences with pain medications, and clinician behaviour in SDM	Recall bias (some interviews conducted months after the trial); single centre
SDM tools for opioid prescribing						
Delara and colleagues,^23^ 2022	RCT	Women undergoing minimally invasive hysterectomy (*n*=73)	Scripted patient-provider conversation	Scripted patient-provider conversation to guide SDM and opioid prescribing decisions	SDM significantly decreased the proportion of unused oxycodone tablets compared with standard care (73.6% *vs* 56.3%; *P*<0.01). When given the choice, patients requested fewer opioid tablets than was standard for our practice at the time of the study, whereas there is no difference in pain control or refill requests.	Single centre; specific surgical population; short-term follow-up
Prabhu and colleagues,^24^ 2017	Prospective quality improvement initiative (single-cohort interventional study)	Women undergoing Caesarean delivery (*n*=50)	Tablet-based decision aid	Tablet-based decision aid presenting information on pain trajectories, opioid risks, and prescription quantity options; patient-selected opioid prescription amount	Median chosen 20 tablets (IQR 15–25) *vs* standard 40 (*P*<0.001); low refill rate; 90% of participants reported being satisfied or very satisfied	Single centre; non-randomised design; small sample size
Vilkins and colleagues,^25^ 2019	Prospective quality improvement study with retrospective comparison group	Women undergoing hysterectomy (post-decision aid *n*=170; 141 completed follow-up; pre-decision-aid comparison group *n*=140)	Visual decision-aid card	Simple visual decision-aid card to facilitate SDM about opioid prescribing after hysterectomy	Significant reduction in opioids prescribed (oral morphine equivalents 92 *vs* 160; *P*<0.001); 76.6% of patients chose less than the maximum; 28% used no opioids; high patient satisfaction	Single centre; specific surgical population; self-reporting bias of opioid use after discharge; limited generalisability
Serna-Gallegos and colleagues,^26^ 2024	RCT (the REPAIR study)	Women undergoing urogynaecological surgery (*n*=154)	Preoperative video education+SDM	Patient-centred preoperative video education combined with SDM for opioid quantity selection	Opioid consumption did not differ between groups (standard, median 3.5 pills [IQR 0–8.25]; patient centred, median 2 pills [IQR 0–9.75]; *P*=0.627); patient-centred group had significantly fewer opioids prescribed (*P*<0.001) and unused (*P*<0.001); no differences in return to function, pain interference, or satisfaction.	Single centre; specific surgical population
Bolson and colleagues,^27^ 2022	RCT	Outpatient hand surgery patients (SDM tool group *n*=25; control group *n*=28)	Voice-over slide presentation+SDM	Voice-over slide presentation on postoperative pain, opioids, and alternatives, combined with SDM	Patients in the SDM tool group were prescribed fewer opioids (mean 6.4 *vs* 10 oxycodone pills; *P*<0.01); opioid consumption was similar between groups (median 2 [IQR 6] *vs* 3 [IQR 6]; *P*=0.97).	Single centre; small sample size; limited generalisability
**Implementation challenges**						
Angelini and colleagues,^28^ 2024	Controlled before-and-after interventional design	Patients undergoing orthopaedic and spine surgery (*n*=98; only 31 of 98 received the complete intervention)	Structured person-centred care intervention	Targeted person-centred care intervention for pain management	No significant improvement in pain relief, patient satisfaction, or SDM; incomplete intervention delivery (only 31 of 98 participants received the full intervention)	Incomplete intervention delivery; organisational barriers; small sample size
van den Berg and colleagues,^29^ 2019	Qualitative study (semi-structured interviews)	Patients undergoing major thoracic or abdominal surgery and healthcare professionals (10 patient interviews; 10 professional interviews)	Prototype digital decision-support tool	Prototype decision-support tool for postoperative analgesia, comparing epidural analgesia with alternative approaches	Patients were minimally involved in decisions and did not express a desire for greater involvement; information provided often promoted epidural analgesia and was not always tailored to individual patient needs or to alternative methods of reducing postoperative pain.	Single centre; prototype tool not validated; small sample
AI-assisted clinical and shared decision making						
Zhang and colleagues,^30^ 2023	Algorithm development and validation study	Cardiothoracic surgical patients (*n*=999)	AI predictive model (Doseformer)	Doseformer: transformer-based AI model using patient characteristics, surgical data, and physiological signals to predict postoperative pain and optimise analgesic dosing	92.16% accuracy for postoperative pain prediction; outperformed baseline models; assisted anaesthetists in identifying 12% more patients with pain-related discomfort	Single centre; prediction errors owing to noninvasive monitoring; requires further validation in an RCT before clinical implementation
Park and colleagues,^31^ 2024	Prospective observational study	Postoperative patients after gastric cancer surgery (*n*=155)	ML facial expression analysis	ML model using facial expression analysis to predict severe postoperative pain intensity	ML model using facial expressions best predicted severe postoperative pain (NRS ≥7) and outperformed models using vital signs alone	Small sample size; static facial expression used (not full video recording); single centre; did not compare performance with validated pain assessment scales
Bellal and colleagues,^32^ 2024	Prospective pilot study	Non-communicating ICU patients (*n*=30)	Automated 3D facial expression AI	Automated 3D facial expression analysis for continuous pain detection	Near 80% sensitivity and 70% specificity for pain detection; insufficient accuracy for pain severity assessment	Small sample size; single centre; temporal inaccuracy (longer time interval to predict pain); requires an RCT to test clinical effectiveness
Ghita and colleagues,^33^ 2023	Proof-of-concept trial	Patients (*n*=12) in the PACU after general anaesthesia	Deep learning model	Mathematical modelling and deep learning classification of pain measured during PACU recovery	Feasible for objective and individualised assessment of pain intensity; comparable performance with clinical pain assessment	Very small sample size; no sensitivity analysis; limited diversity; requires labelled dataset for model training
Gabriel and colleagues,^34^ 2023	Retrospective cohort study	Patients undergoing ambulatory surgery (*n*=1333)	ML algorithm (opioid refill prediction)	Four ML algorithms (neural network, logistic regression, random forest, and support vector classifier) to predict likelihood of opioid prescription refill after surgery	10.8% of patients refilled their opioid prescription within 2 weeks; active smoking history, intraoperative and PACU opioid use, regional nerve block use, and PACU pain ratings were the strongest predictors; approach identifies high-risk patients for early referral to specialised care.	Retrospective design; single centre
Wang and colleagues,^35^ 2023	Retrospective observational study	Postoperative patients managed with AI-PCA (2018–2021; *n*=14 747); historical control group without AQI guidance (2014–2018)	AI-PCA)	AI-PCA with AQI optimisation	Reduced incidence of moderate-to-severe pain (21.7% *vs* 26.3% in historical control group); improved overall pain management; integration of the technology into clinical workflows was complex	Single centre; retrospective design; no randomised comparison

## Shared decision making: from personalisation to digital implementation

SDM in acute pain management enables patients to actively collaborate with clinicians in selecting among treatment options, rather than solely following clinician-directed plans.^15^ These options may include multimodal non-opioid analgesic regimens and non-pharmacological interventions. Decision-support tools have been studied in the context of acute pain management. These tools can take many forms, both digital and non-digital, including scripted conversations between patients and providers, tablet-based decision aids, single-page graphic cards, and video narratives. The complexity, required resources, and level of patient involvement differ across these formats. Currently, there are no direct comparative studies demonstrating that one tool format is superior to others; the most effective design likely depends on the clinical setting, patient population, and available resources. In addition, there remains a significant gap in evidence regarding SDM tools that address the full range of multimodal analgesic options beyond just opioid prescribing.

## Clinician-supported shared decision making

Some studies have examined how patient-clinician collaboration can support the implementation of SDM in acute pain management. For instance, Manoharan and colleagues^20^ developed a personalised acute pain service based on SDM principles, called the personalised pain programmes. Their approach emphasised building strong therapeutic relationships and trust through transitional perioperative pain services, which provided specialised pain management and simultaneous psychiatric treatment for patients with co-morbid mental health disorders, such as depression and anxiety.

This service also aimed at supporting patients with complex medical histories, psychological distress, and preoperative opioid use. Participants valued the SDM process, which nurtured a trusting relationship with their physician, set clear pain expectations, and delivered personalised care. They also benefited from access to both psychiatry and pain specialists. Compared with standard care, participants reported reduced opioid consumption and improved recovery after surgery, linked to their positive experiences.

Although promising, the personalised pain programme is resource intensive and may be challenging to implement. These findings suggest that SDM can be facilitated through both multidisciplinary pain services and relatively simple structured conversations. However, institutional capability remains a limiting factor in both cases.

## Digital decision aids

Recently, digital and interactive decision aids have been introduced for acute pain management in various hospital settings to enhance patient engagement. Engel-Rebitzer and colleagues^21^ found that video narratives about opioids, featuring storytellers who matched the patients in race or gender, improved patients’ ability to recall information about pain management options the day after discharge compared with those who viewed videos without culturally matched storytellers. Videos that addressed family histories of addiction also increased patient participation in treatment decisions. However, the helpfulness of pain treatment information reported by patients, their preferences for opioids, and satisfaction with pain management did not correlate with the specific narrative elements examined in the videos.^21^

In addition, Dolan and colleagues^22^ conducted a qualitative study nested within an RCT to explore patients’ responses to two opioid risk communication tools: a probabilistic opioid risk tool and a narrative-enhanced probabilistic risk tool (short video stories). These tools prompted meaningful reflections from patients about their choices regarding pain treatment.^22^ The success of these tools depended on various factors influencing the SDM process, including self-identity, personal beliefs, previous experiences with pain medications, clinicians’ behaviour, biases, and experiences of discrimination. These findings are consistent with previous evidence indicating that perioperative factors, such as past patients’ experiences with opioid use, their beliefs, and clinician communication, affect postoperative behaviour.^9^

Several studies have evaluated simple digital and structured tools to guide SDM for opioid prescribing after surgery. Delara and colleagues^23^ used a scripted patient-provider conversation to facilitate the SDM process and determine the number of prescribed oxycodone tablets after a minimally invasive hysterectomy. This approach significantly reduced the amount of unused oxycodone tablets without impairing access to opioids. Prabhu and colleagues^24^ tested a tablet computer-based decision aid to guide opioid prescribing after Caesarean delivery. This decision aid provided information on expected pain trajectories, opioid risks and benefits, and non-opioid alternatives and included a visual analogue scale for patients to indicate their preferred prescription quantity. As a result, the number of prescribed oxycodone tablets was reduced by approximately half, the refill rate was low (8%), and 90% of participants reported being satisfied or very satisfied with their outpatient pain management.

In another successful implementation of SDM, Vilkins and colleagues^25^ used a straightforward, visual decision-aid card to facilitate SDM regarding opioid prescribing after hysterectomy. This single-page card presented information on typical pain patterns, opioid risks, non-opioid options, and prescription quantities. The study indicated a significant reduction in prescribed opioids, with 76.6% of participants opting for less than the maximum amount and 28% using none. Patient satisfaction remained high, refill requests did not increase, and 76% reported having leftover tablets.

Educational video interventions combined with SDM also improved prescribing efficiency and reduced leftover opioids in urogynaecological and hand surgery.^26^^,^^27^ The intervention in Serna’s study^26^ combined patient-centred preoperative video education (which covered postoperative pain expectations, opioid risks, and multimodal analgesia options) with SDM, allowing patients to select their discharge oxycodone quantity. Although opioid consumption was comparable between the standard-care and patient-centred groups, those in the patient-centred group were prescribed significantly fewer opioid pills and had fewer unused pills left. No differences in return to function, pain interference, preparedness, or satisfaction were found. A similarly effective tool was a short voice-over slide presentation on postoperative pain, opioids, and alternative treatments implemented by Bolson and colleagues.^27^ This educational SDM tool was designed to empower patients and individualise pain management, ultimately reducing opioid prescriptions while maintaining similar oxycodone consumption rates and pain control.

These results suggest that digital decision aids enhance patient engagement and understanding while reducing opioid prescriptions without compromising pain control or patient satisfaction. However, their effectiveness depends not only on the delivery of information but also on patients’ perceptions, previous experiences, and interactions with their physicians.

## Shared decision making implementation challenges

Despite promising results from individual studies, the implementation of SDM in acute pain management faces significant challenges. In orthopaedics and spine surgery, Angelini and colleagues^28^ found that a targeted person-centred care intervention did not improve postoperative pain relief, patient satisfaction, or SDM. Incomplete and inconsistent implementation was likely a factor, given that only 31 of 98 participants received the full intervention. In addition, issues such as organisational pressure, staff turnover, and patient relocation negatively affected the implementation process.

Similarly, van den Berg and colleagues^29^ investigated preoperative SDM and a prototype decision-support tool for postoperative analgesia after major thoracic or abdominal surgery. They found that patients were minimally involved in decision making and often did not express a desire for greater involvement; many relied on their physicians’ expertise by default. The information provided mostly focused on epidural analgesia and was not always tailored to meet patients’ specific needs or to explore other methods of reducing postoperative pain. Although patients viewed the decision-support tool positively owing to its clarity, they felt that it would not have changed their decisions. The professionals involved in this study questioned patients’ ability to fully understand the choices they were making.

## Future implementation

Even though evidence from chronic pain management can enhance our understanding of acute pain management, more research is needed to assess its relevance to perioperative care requires further study. SDM has the potential to strengthen opioid stewardship programmes in acute care, emphasising pain management and involving pharmacists.^36^ Pharmacists can play a crucial role in educating both patients and clinicians, identifying misuse, developing treatment plans, assisting with tapering, and promoting naloxone use.^12^^,^^36^ This highlights the essential need for collaboration between secondary and primary care.

However, several obstacles hinder the implementation of SDM, including limited time, inadequate collaboration with prescribers, some patients’ reluctance to engage in the process, and systemic barriers to integration. There are various structured tools available in acute care settings to support participatory decision making using simple approaches. These include a five-step process consisting of seeking patient participation, helping patients explore and compare treatment options, assessing their values and preferences, reaching a decision together, and evaluating the patient’s decision; making good decisions in collaboration; benefits, risks, alternatives, and what happens if nothing is done; and multifocal approach to sharing in SDM.^14^

Overall, if SDM is to be used effectively, addressing the constraints of limited resources, support, and training is essential.^14^ Comprehensive strategies may involve multidisciplinary approaches (engaging various medical disciplines), multiprofessional collaboration (including nursing and pharmacy), and systemic initiatives (such as guideline development and implementation, education, and feedback for clinicians). In addition, pragmatic, simplified, or even automated decision-support tools could help remove these barriers.

## The role of artificial intelligence in enhancing clinical and shared decision making

Although SDM provides patients with the information needed to participate in their care, the quality of these discussions depends significantly on the robustness of the clinical evidence that supports them. AI is increasingly being explored as a tool to enhance this evidence base, leading to more accurate pain predictions, objective assessments, and personalised analgesic strategies. However, AI should not be seen as a replacement for the patient-clinician dialogue essential for SDM; instead, it should serve as a decision-support tool that enhances and informs this dialogue.

As’ad and colleagues^37^ have proposed a conceptual framework called AI-SDM, which integrates predictive modelling with generative AI reasoning to support SDM. This framework translates algorithmic outputs, such as predicted pain scores, opioid risk estimates, or personalised analgesic recommendations, into structured conversations between clinicians and patients, all while preserving human judgement and patient autonomy.

A predictive tool has been suggested to improve the prediction of postoperative pain and the use of pain management techniques.^37^ Although preliminary results were encouraging, limited performance at the individual level may limit its potential.^30^ Alongside prediction, AI has also been proposed as a tool to improve objective assessment of acute pain after surgery.^31^ Relevant attempts have also been made with non-communicating intensive care patients using automated decision-support tools and continuous three-dimensional analysis of facial expressions. Although the performance of these tools in detecting pain-related behaviours was noteworthy, their output, such as pain intensity estimates and assessment scores, was not reliable enough to conclusively assess the severity of pain. It reinforces the assumption that decision-support tools’ results must be integrated into a communication plan with patients, a core principle of SDM.^32^

Mathematical modelling and deep learning for individualised pain intensity assessment were tested in the PACU using the physiological fractional-order pragmatic model and spectrographic-based convolutional neural networks.^33^ Machine learning algorithms have also been used to predict the need for opioid refills after surgery in a retrospective, single-centre study.^34^ The correlation observed across initial opioid intake, pain score patterns (i.e. median and maximum pain ratings), and opioid refills suggests that AI tools may enrich, in the future, SDM discussions.^34^ If these findings can be replicated in prospective studies and other settings, the potential for improving care is substantial. For example, if refill risk can be anticipated at discharge, clinicians and patients can collaboratively plan analgesic strategies, manage expectations, and agree on a follow-up plan, directly applying SDM principles to opioid stewardship.

AI-enhanced patient-controlled analgesia (AI-PCA) may represent an advanced approach to empowering patients in acute pain management. AI-PCA combines traditional PCA with the Internet of Things and AI, linking the analgesia pump to a central computer.^35^^,^^38^ This enables remote monitoring and assessment and the calculation of the Analgesia Quality Index (AQI) using real-time parameters, including the frequency of patient-controlled button presses, patient information, alarm history, and drug use over a defined period. These data can be used to optimise postoperative analgesia. This retrospective observational study compared the outcomes of AQI in postoperative pain management from 2018 to 2021 with a control group that did not receive AQI guidance from 2014 to 2018.^35^ Although the AQI was associated with improvements, insufficient analgesia levels were observed more frequently in the AQI management group, and the integration of this technology proved complex.^35^ Notably, a survey of anaesthesia and medical staff in China revealed that although the staff had good knowledge and positive attitudes towards AI-PCA, their application of it was inadequate, highlighting the need for structured training programmes.^39^ AI-PCA exemplifies the collaboration between AI and SDM, where patients maintain control over their analgesia through PCA mechanisms, whereas AI provides clinicians with objective, real-time quality metrics to guide adjustments.

Despite significant promise, the development of AI applications in acute pain management is still in its early stages. The existing evidence base is limited, given that most studies are characterised by small sample sizes, single-centre design, and retrospective methodology. None of the AI tools mentioned have been validated across diverse patient groups and clinical environments. In addition, staff training and institutional infrastructure are essential for the integration of AI into clinical practice.^40^

For AI to genuinely enhance SDM, these tools must be explainable and integrated into care processes that prioritise the physician’s role, rather than allowing algorithms to dominate decision making. Furthermore, careful evaluation of AI systems is necessary to identify and address any algorithmic bias and to ensure equitable performance across diverse patient populations before implementation.

Although there is cautious optimism surrounding these concepts, they must be rigorously validated in a variety of settings to truly enhance clinical accuracy and enrich conversations between patients and clinicians.

## Conclusion

Herein, we provide a concise overview of the growing evidence supporting the use of digital health technologies and AI to promote SDM and transform acute pain management into a more patient-centred process. Digital decision aids, structured communication tools, and AI-supported methods show promise in optimising pain relief and addressing concerns related to opioid use. However, the current evidence remains heterogeneous, largely derived from single-centre studies, and primarily focuses on elective surgeries. There are limited data on SDM approaches that encompass the broader spectrum of multimodal analgesics, not just opioids.

AI and machine learning offer meaningful potential for predicting and assessing pain but face major challenges related to validation across different patient groups and clinical settings and issues related to training and resource requirements.

To translate these advances into practical applications, it is essential to go beyond merely developing technology; we must foster institutional commitment to training, equity, and patient engagement. SDM must extend beyond opioid prescriptions and should also include a variety of analgesic options. Furthermore, it should prioritise patients’ goals of functional recovery and quality of life, rather than solely focusing on pain intensity scores.

Implementation should start with straightforward, low-resource interventions such as scripted conversations, single-page decision aids, and structured frameworks, eventually scaling up to more complex digital systems tailored to specific clinical contexts and patient populations. Multiprofessional collaboration is essential: pharmacists, pain specialists, mental health providers, and primary care teams each play vital roles in supporting SDM throughout care transitions. Training programmes must equip clinicians not only with the technical skills to use AI tools but also with the communication skills needed to translate algorithm outputs into meaningful dialogues with patients, ensuring that human oversight and clinical judgement remain paramount.

Moving forward, a collaborative strategy that prioritises patient safety, effectiveness, and overall well-being is essential. By working with patients as partners, we can leverage digital health technologies and AI tools to promote SDM in acute pain management. Ultimately, the success of these tools depends on embedding them within a culture of open communication, equity, and shared purpose, ensuring that patients’ values are meaningfully reflected in the decisions regarding their care.

## Authors’ contributions

Conceptualisation of the study: all authors

Writing—original draft: DMA, SSA

Writing, review, and editing: PF

Reviewed and approved the final version of this article and have read and confirmed meeting the ICMJE criteria for authorship: all authors

## Declaration of interests

PF is supported by the European Society of Anaesthesiology and Intensive Care (ESAIC) for the Pain and Opioids After Surgery and the Euro-Periscope Research Groups (IDs ESAIC_GR_2021_PF, ESAIC_RG_PAND, and ESAIC_RG_EP) and received advisory board/speaker fees from Grünenthal, GE HealthCare, and Fresenius Kabi. DMA and SSA are Saudi Government Scholarship holders (PhD) sponsored by Prince Sultan Military College for Health Sciences, Health Services General Directorate, Ministry of Defence, Kingdom of Saudi Arabia.
